# An Essay on Mercurial Fumigations

**Published:** 1829-05

**Authors:** Jonathan Green

**Affiliations:** Surgeon.


### MERCURIAL FUMIGATIONS.

*An Essay on Mercurial Fumigations.* By Jonathan Green, Surgeon.

Vapour baths, by far the most powerful of all sudorific remedies, are so effectual in the removal of some of the various forms of syphilitic disease, that authors have attributed the infrequency and mildness of these affections in eastern countries to their constant use. In the treatment of syphilis, they may be employed as auxiliary and preparatory to, or at the same time with, mercury internally; and, both after and during the administration of vapour baths, this specific remedy is found to act with greater promptness and efficacy, removing affections of long standing, which had even previously obstinately resisted its employment.

In those cases (of too frequent occurrence) wherein, for apparently local symptoms, a greater quantity of mercury has been administered than necessary by the usual methods, and yet the virus is not extinguished, the simple vapour baths have been employed with the most marked success, and, without other assistance, have perfected the cure. They continually tend to diminish the violence of the disease; they regulate its progress, and abridge its duration.

When vapour baths are employed as the principal remedy, they are administered under the form of mercurial fumigations; and experience has amply shown the advantages of mercury under the form of vapour in the treatment of syphilis. On the first appearance of this disease in Europe, towards the end of the fifteenth century, the analogy which was thought to exist between it and certain cutaneous affections, against which mercurial fumigations had been long and successfully directed, led to the adoption of the same means in it, which thus became the first antisyphilitic method of treatment, after the aromatic fumigations, then generally in use, had been tried in vain; and had they at that time possessed the perfect means of administering them which are now in our power, they would probably have known no other. But the different modes of proceeding then in use were so imperfect, and subject to such serious inconveniences, that they found themselves obliged to substitute frictions in its stead; which were also administered, at the beginning, in so very defective a manner, that they were productive of as dangerous results.

Another specific was then anxiously sought after, and thought to have been found in the sudorific woods; but experience soon placed them in the rank of auxiliary remedies, where they have ever since remained; although very remarkable cures have certainly been effected by their influence alone. To the real specific, mercury, then, it became necessary to return, and it was now tried internally under various forms; and then to the two other was added a third antisyphilitic method, much more dangerous than either.

Frictions, however, were afterwards employed with more method, and consequently with greater success; while at the same time fruitless efforts were made to bring to perfection the method of administering mercurial vapour, the advantages whereof were still too highly appreciated to permit of their total abandonment.

Thus, for the long space of three centuries that mercurial fumigations have been frequently resorted to, the little attention given to their employment, and the defective state of the fumigating apparatus, have often disappointed the hopes of the practitioner.

Towards the end of the last century, Lallonette, a physician of Paris, used them with great success, and published a work containing many instances of syphilitic disease which, having resisted all other means, were perfectly restored by this method; and he has given the best instructions and rules for their employment that have yet appeared. It would seem, however, that his great success was not so much owing to the excellence of his apparatus, which, although superior to all former ones, was still very imperfect, as to the care and attention he bestowed on their administration, and to the preparations from which he disengaged the vapour having none of the inconveniences of those that were previously employed. The advantages, in the treatment of syphilis, to be derived from the employment of the mercurial vapour in fumigations to the whole surface of the body, or any part thereof, are incalculable, now that we possess such sure and convenient means for exhibiting them. Mercurial fumigations excite, with extreme facility, both those effects, without which syphilis cannot be cured, the absorption of the mercury and free perspiration. They neither require nor operate to the exclusion, at least in the greater number of instances, of the employment of any auxiliary means. They may be used, with the utmost safety, at all seasons of the year, demanding no precaution or particular attention whatever, beyond the time of their administration; and, so far from weakening the patient, his strength is increased under the influence of this method of fumigating, which may be persisted in uninterruptedly to the perfect extinction of all the symptoms. This method is the only one to which recourse may be had without danger in pregnant women, nurses, and infants. It also gives to patients a facility of concealing from every body a knowledge of their situation; while, in following other methods, they are always obliged to admit one or more persons into their confidence; and in this manner it is evident that many inconveniences, and disturbances, and divisions in families, may be averted by the employment of mercurial fumigations.

As a preventive against syphilis, the mercurial fumigations, it is reasonable to conclude, may be superior to all others; for, being used immediately or soon after the infection, it should annul its effects by the complete absorption of the mercurial vapour, which is spread over the whole surface in a state of infinite divisibility. The progress of the contagion will be arrested, and the principle of the disease extinguished, before it has had time to affect the constitution. Thus it seems probable that, by the use of a few mercurial fumigations after infection, the disease will not only be prevented from developing itself, but the very source from which it would have proceeded will be dried up.

In fine, so generally effectual are mercurial fumigations in syphilis, and at the same time so perfectly safe, that some French physicians, well acquainted with these facts from their own experience, have conceived the philanthropic idea of exterminating this scourge of the human race by means of their universal application, to be effected by the formation of establishments in every town, to which all the infected may be indiscriminately admitted.

40, *Great Marlborough street; April* 1829.

